# Redetermination of Sr_2_PdO_3_ from single-crystal X-ray data

**DOI:** 10.1107/S2056989018017176

**Published:** 2019-01-01

**Authors:** Gohil S. Thakur, Hans Reuter, Claudia Felser, Martin Jansen

**Affiliations:** a Max Planck Institut for Chemical Physics of Solids, Nöthnitzer Straβe 40, 01187, Dresden, Germany; b Institute for Chemistry of New Materials, University of Osnabrück, Barbarastrasse, 7, 49076 Osnabrück, Germany; c Max Planck Institut for Solid State Research, Heisenbergstr. 1, 70569 Stuttgart, Germany

**Keywords:** crystal structure, K_2_NiF_4_ structure, Sr_2_CuO_3_ structure type, linear chain compound

## Abstract

The crystal structure redetermination of Sr_2_PdO_3_ (distrontium palladium trioxide) was carried out using high-quality single-crystal X-ray data. The Sr_2_PdO_3_ structure has been described previously in at least three reports [Wasel-Nielen & Hoppe (1970 ▸). *Z. Anorg. Allg. Chem*. **375**, 209–213; Muller & Roy (1971 ▸). *Adv. Chem. Ser*. **98**, 28–38; Nagata *et al.* (2002 ▸). *J. Alloys Compd.* **346**, 50–56], all based on powder X-ray diffraction data. The current structure refinement of Sr_2_PdO_3_, as compared to previous powder data refinements, leads to more precise cell parameters and fractional coordinates, together with anisotropic displacement parameters for all sites. The compound is confirmed to have the ortho­rhom­bic Sr_2_CuO_3_ structure type (space group *Immm*) as reported previously. The structure consists of infinite chains of corner-sharing PdO_4_ plaquettes inter­spersed by Sr^II^ atoms. A brief comparison of Sr_2_PdO_3_ with the related K_2_NiF_4_ structure type is given.

## Abstract 2

Sr_2_PdO_3_ adopts the Sr_2_CuO_3_ structure type. In comparison with previous determinations, the present redetermination results in improved precision of the structural parameters.

## Chemical context   

Low-dimensional transition-metal oxides with chain structures have received attention since they can enable inter­esting physical phenomena such as spin 1/2 anti­ferromagnetic Heisenberg coupling (Motoyama *et al.*, 1996 ▸; Takigawa *et al.*, 1996 ▸), superconductivity (Hiroi *et al.*, 1993 ▸), ultrafast non-linear optical response (Ogasawara *et al.*, 2000 ▸) or even glucose sensing (El-Ads *et al.*, 2016 ▸). The particularly relevant sub-family based on square-planar *M*O_4_ (*M* = divalent metal) primary building units is dominated by oxidocuprates(II), while the chemistry of respective palladates(II), showing the same preference for a square-planar coordination by oxygen, is much less explored.

Here we address Sr_2_PdO_3_, which has previously been obtained as a microcrystalline material (Wasel-Nielen & Hoppe, 1970 ▸; Muller & Roy, 1971 ▸; Nagata *et al.*, 2002 ▸). Based on evaluations of powder X-ray diffractograms, Sr_2_PdO_3_ was identified as being isostructural with Sr_2_CuO_3_ (Teske & Müller-Buschbaum, 1969 ▸; Weller & Lines, 1989 ▸) and Sr_2_FeO_3_ (Tassel *et al.*, 2013 ▸). However, structural details derived from the given atomic parameters have only been reported with large uncertainties (Muller & Roy, 1971 ▸; Nagata *et al.*, 2002 ▸). Therefore, a redetermination of Sr_2_PdO_3_ based on single crystal X-ray data seemed appropriate.

## Structural commentary   

**Figure 1:**
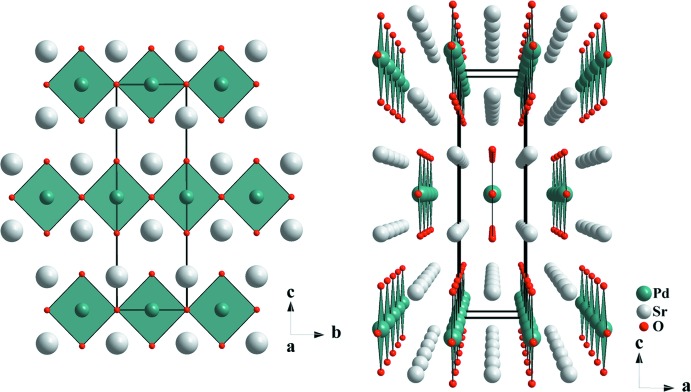
Crystal structure of Sr_2_PdO_3_ viewed along the *a* axis (left) and along the *b* axis (right).

**Figure 2:**
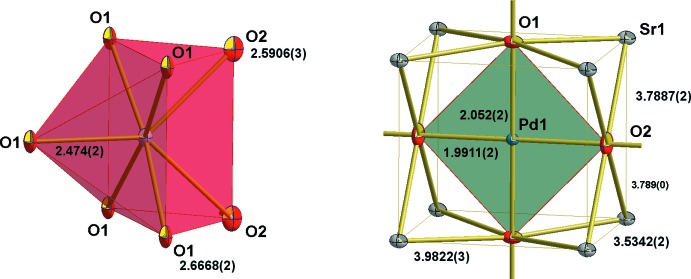
Coordination around the Sr^II^ (left) and Pd^II^ atoms (right). All atoms are drawn with displacement ellipsoids at the 80% probability level. Distances are in Å.

**Figure 3:**
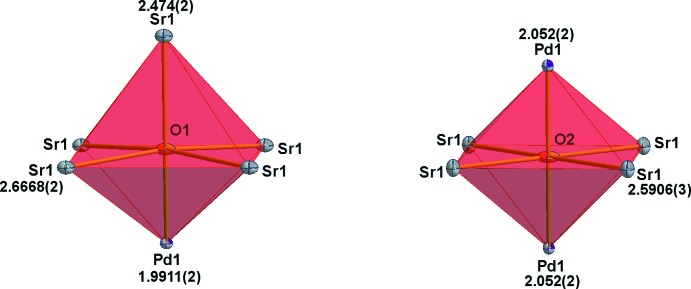
Coordination polyhedra of two types of oxygen atoms, O1 (left) and O2 (right). All atoms are drawn with displacement ellipsoids at the 80% probability level. Distances are in Å.

The crystal structure of Sr_2_PdO_3_ is essentially the same as determined previously (Wasel-Nielen & Hoppe, 1970 ▸; Muller & Roy, 1971 ▸; Nagata *et al.*, 2002 ▸). The lattice parameters (Table 1 ▸) are almost identical to those in the previous reports but with higher precision. The Pd^II^ atom occupies the 2*d* crystallographic sites with *mmm* site symmetry. We would like to point out that we chose a different cell setting as compared to all the previous reports, where the Pd^II^ atom was chosen to be located at the cell origin (site 2*a*; 0, 0, 0; hence the different site designations). The Pd^II^ atom forms distorted PdO_4_ square planes, which are linked by sharing oxygen atoms in the *trans*-position to form infinite chains extending along the *b-*axis direction as shown in Fig. 1 ▸. Corresponding to this connectivity pattern, the Pd—O bond lengths are longer for the shared oxygen atoms, 2.052 (2) Å, and shorter for the terminal ones, 1.9911 (2) Å. The Sr atom is situated at the 4*j* Wyckoff site having *mm*2 site symmetry. It is seven-coordinate in a monocapped trigonal–prismatic fashion by oxygen with three different bond lengths (Table 1 ▸, Fig. 2 ▸). In addition to the square-planar first coordination of Pd^II^ with oxygen, the second consists of eight Sr^II^ atoms present at the corner of a cuboid with dimension 3.5342 (2) × 3.7887 (2) × 3.9822 (3) Å^3^ (Fig. 2 ▸). Of the two kinds of oxygen atoms, both surrounded by six metal ions that form distorted octa­hedra, O1 is coordinated by one Pd^II^ atom [2.052 (2) Å] and five Sr^II^ atoms with one short [2.474 (2) Å] and four long distances [2.6668 (2) Å] (Fig. 3 ▸). O2 is connected to four equidistant Sr^II^ [2.5906 (3) Å] and two Pd^II^ atoms [1.9911 (2) Å] (Fig. 3 ▸). In our current structure determination, much more precise values of the cell parameters along with the *z* parameters of Sr and O1 have been determined, consequently, yielding very precise values for the bond lengths (see Table 1 ▸). The quality of the current refinement is also clearly reflected by better reliability factors (see Table 2 ▸) as compared to the previous refinements. The atomic arrangement described here is same as provided by Wasel-Nielen & Hoppe (1970 ▸).

**Figure 4:**
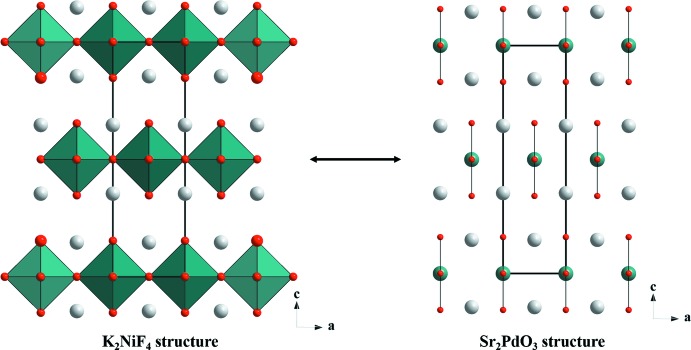
Inter­conversion of the Sr_2_PdO_3_ and K_2_NiF_4_ structures.

The structural features discussed above are closely related to those of the K_2_NiF_4_ type of structure, which is regarded as the prototype structure for all the high *T_c_* cuprates. K_2_NiF_4_ consists of layers of corner-shared NiF_6_ octa­hedra extending in the *ab* plane. One can derive the Sr_2_PdO_3_ structure from the K_2_NiF_4_ structure by systematically removing the bridging F atoms from the NiF_6_ octa­hedra lying in the *a*-axis direction (Fig. 4 ▸). This would reduce the dimensionality of the layer, resulting in linear chains of square planes connected by edges along only one direction.

## Synthesis and crystallization   

Millimeter-sized block-shaped crystals of dark-yellow colour with composition Sr_2_PdO_3_ as confirmed by SEM–EDS, were obtained from a mixture of different phases while attempting to synthesize SrPd_3_O_4_ using a KOH flux (Smallwood *et al.*, 2000 ▸). SrCO_3_ and Pd metal powder were mixed in the molar ratio of 2:3, placed in an alumina crucible, and 15 grams of KOH pellets were added on top. The crucible was heated in a muffle furnace to 1023 K in 24 h with a 6 h dwell time. The furnace was then cooled slowly to 873 K over 125 h after which it was switched off and allowed to cool naturally. The product was washed several times with water to remove the solidified flux and subsequently rinsed with ethanol.

## Refinement   

Crystal data, data collection and structure refinement details are summarized in Table 2 ▸.

## Supplementary Material

Crystal structure: contains datablock(s) I. DOI: 10.1107/S2056989018017176/wm5474sup1.cif

Structure factors: contains datablock(s) I. DOI: 10.1107/S2056989018017176/wm5474Isup2.hkl

CCDC reference: 1882781

Additional supporting information:  crystallographic information; 3D view; checkCIF report

## Figures and Tables

**Table 1:** Comparison of lattice parameters and bond lengths (Å) in Sr_2_PdO_3_ determined in different studies

	1970 work^*a*^	1971 work^*b*^	2002 work^*c*^	This work
*a*	3.977	3.97	3.985	3.5342 (2)
*b*	3.53	3.544	3.539	3.9822 (3)
*c*	12.82	12.84	12.847	12.8414 (8)
Pd—O1 (×2)	2.08	2.045	2.068	2.052 (2)
Pd—O2 (×2)	1.99	1.985	1.993	1.9911 (1)
Sr—O1	2.45	2.504	2.467	2.474 (2)
Sr—O1 (×4)	2.67	2.668	2.671	2.6668 (2)
Sr—O2 (×2)	2.58	2.57	2.588	2.5906 (3)

References: (*a*) Wasel-Nielen & Hoppe (1970 ▸); (*b*) Muller & Roy (1971 ▸); (*c*) Nagata *et al.* (2002 ▸).

**Table 2:** Experimental details

Crystal data	
Chemical formula	Sr_2_PdO_3_
*M*_r_	329.64
Crystal system, space group	Orthorhombic, *I* *m* *m* *m*
Temperature (K)	296
*a*, *b*, *c* (Å)	3.5342 (2), 3.9822 (3), 12.8414 (8)
*V* (Å^3^)	180.73 (2)
*Z*	2
Radiation type	Mo *K*α
μ (mm^−1^)	34.15
Crystal size (mm)	0.18 × 0.16 × 0.12

Data collection	
Diffractometer	Bruker APEXII CCD
Absorption correction	Multi-scan (*SADABS*; Bruker, 2009 ▸)
*T*_min_, *T*_max_	0.062, 0.102
No. of measured, independent and observed [*I* > 2σ(*I*)] reflections	8304, 178, 176
*R*_int_	0.035
(sin θ/λ)_max_ (Å^−1^)	0.702

Refinement	
*R*[*F* ^2^ > 2σ(*F* ^2^)], *wR*(*F* ^2^), *S*	0.009, 0.021, 1.27
No. of reflections	178
No. of parameters	16
Δρ_max_, Δρ_min_ (e Å^−3^)	0.43, −0.51

Computer programs: *APEX2* and, *SAINT* (Bruker, 2009 ▸), *SHELXS97* and *SHELXTL* (Sheldrick, 2008 ▸), *SHELXL2014/7* (Sheldrick, 2015 ▸), *DIAMOND* (Brandenburg, 2006 ▸).

## supplementary crystallographic information

### Crystal data

**Table:**

Sr_2_PdO_3_	*D*_x_ = 6.057 Mg m^−^^3^
*M**_r_* = 329.64	Mo *K*α radiation, λ = 0.71073 Å
Orthorhombic, *I**m**m**m*	Cell parameters from 1490 reflections
*a* = 3.5342 (2) Å	θ = 3.2–29.9°
*b* = 3.9822 (3) Å	µ = 34.15 mm^−^^1^
*c* = 12.8414 (8) Å	*T* = 296 K
*V* = 180.73 (2) Å^3^	Block, yellow-brown
*Z* = 2	0.18 × 0.16 × 0.12 mm
*F*(000) = 292	

### Data collection

**Table:**

Bruker APEXII CCD diffractometer	176 reflections with *I* > 2σ(*I*)
φ and ω scans	*R*_int_ = 0.035
Absorption correction: multi-scan (*SADABS*; Bruker, 2009)	θ_max_ = 29.9°, θ_min_ = 3.2°
*T*_min_ = 0.062, *T*_max_ = 0.102	*h* = −4→4
8304 measured reflections	*k* = −5→5
178 independent reflections	*l* = −18→18

### Refinement

**Table:**

Refinement on *F*^2^	0 restraints
Least-squares matrix: full	*w* = 1/[σ^2^(*F*_o_^2^) + (0.0092*P*)^2^ + 0.2817*P*] where *P* = (*F*_o_^2^ + 2*F*_c_^2^)/3
*R*[*F*^2^ > 2σ(*F*^2^)] = 0.009	(Δ/σ)_max_ < 0.001
*wR*(*F*^2^) = 0.021	Δρ_max_ = 0.43 e Å^−^^3^
*S* = 1.27	Δρ_min_ = −0.51 e Å^−^^3^
178 reflections	Extinction correction: SHELXL-2014/7 (Sheldrick, 2015), Fc^*^=kFc[1+0.001xFc^2^λ^3^/sin(2θ)]^-1/4^
16 parameters	Extinction coefficient: 0.0059 (5)

### Special details

**Table:**

Geometry. All esds (except the esd in the dihedral angle between two l.s. planes) are estimated using the full covariance matrix. The cell esds are taken into account individually in the estimation of esds in distances, angles and torsion angles; correlations between esds in cell parameters are only used when they are defined by crystal symmetry. An approximate (isotropic) treatment of cell esds is used for estimating esds involving l.s. planes.

### Fractional atomic coordinates and isotropic or equivalent isotropic displacement parameters (Å^2^)

**Table:**

	*x*	*y*	*z*	*U*_iso_*/*U*_eq_	
Pd1	0.5000	0.0000	0.5000	0.00493 (11)	
Sr1	0.5000	0.0000	0.14752 (2)	0.00656 (11)	
O1	0.5000	0.0000	0.34021 (18)	0.0085 (5)	
O2	0.5000	0.5000	0.5000	0.0128 (8)	

### Atomic displacement parameters (Å^2^)

**Table:**

	*U*^11^	*U*^22^	*U*^33^	*U*^12^	*U*^13^	*U*^23^
Pd1	0.00765 (19)	0.00396 (17)	0.00320 (18)	0.000	0.000	0.000
Sr1	0.00752 (17)	0.00760 (16)	0.00456 (17)	0.000	0.000	0.000
O1	0.0117 (13)	0.0101 (12)	0.0037 (11)	0.000	0.000	0.000
O2	0.021 (2)	0.0035 (15)	0.0134 (18)	0.000	0.000	0.000

### Geometric parameters (Å, º)

**Table:**

Pd1—O2^i^	1.9911 (1)	Sr1—O1^ix^	2.6668 (2)
Pd1—O2	1.9911 (1)	Sr1—O1^vii^	2.6668 (2)
Pd1—O1	2.052 (2)	Sr1—Pd1^xi^	3.2674 (2)
Pd1—O1^ii^	2.052 (2)	Sr1—Pd1^xii^	3.2674 (2)
Pd1—Sr1^iii^	3.2674 (2)	Sr1—Pd1^xiii^	3.2674 (2)
Pd1—Sr1^iv^	3.2674 (2)	Sr1—Pd1^xiv^	3.2674 (2)
Pd1—Sr1^v^	3.2674 (2)	Sr1—Sr1^xv^	3.5342 (2)
Pd1—Sr1^vi^	3.2674 (2)	O1—Sr1^viii^	2.6668 (2)
Pd1—Sr1^vii^	3.2674 (2)	O1—Sr1^iii^	2.6668 (2)
Pd1—Sr1^viii^	3.2674 (2)	O1—Sr1^vii^	2.6668 (2)
Pd1—Sr1^ix^	3.2674 (2)	O1—Sr1^ix^	2.6668 (2)
Pd1—Sr1^x^	3.2674 (2)	O2—Pd1^xvi^	1.9911 (1)
Sr1—O1	2.474 (2)	O2—Sr1^ix^	2.5906 (3)
Sr1—O2^xi^	2.5906 (3)	O2—Sr1^iv^	2.5906 (3)
Sr1—O2^xii^	2.5906 (3)	O2—Sr1^viii^	2.5906 (3)
Sr1—O1^viii^	2.6668 (2)	O2—Sr1^vi^	2.5906 (3)
Sr1—O1^iii^	2.6668 (2)		
			
O2^i^—Pd1—O2	180.0	O1—Sr1—O1^vii^	86.61 (5)
O2^i^—Pd1—O1	90.0	O2^xi^—Sr1—O1^vii^	119.68 (4)
O2—Pd1—O1	90.0	O2^xii^—Sr1—O1^vii^	65.87 (4)
O2^i^—Pd1—O1^ii^	90.0	O1^viii^—Sr1—O1^vii^	96.597 (9)
O2—Pd1—O1^ii^	90.0	O1^iii^—Sr1—O1^vii^	83.000 (8)
O1—Pd1—O1^ii^	180.0	O1^ix^—Sr1—O1^vii^	173.22 (10)
O2^i^—Pd1—Sr1^iii^	52.455 (3)	O1—Sr1—Pd1^xi^	125.435 (5)
O2—Pd1—Sr1^iii^	127.545 (3)	O2^xi^—Sr1—Pd1^xi^	37.546 (3)
O1—Pd1—Sr1^iii^	54.565 (5)	O2^xii^—Sr1—Pd1^xi^	86.845 (8)
O1^ii^—Pd1—Sr1^iii^	125.435 (5)	O1^viii^—Sr1—Pd1^xi^	147.95 (5)
O2^i^—Pd1—Sr1^iv^	127.545 (3)	O1^iii^—Sr1—Pd1^xi^	38.82 (5)
O2—Pd1—Sr1^iv^	52.455 (3)	O1^ix^—Sr1—Pd1^xi^	97.52 (3)
O1—Pd1—Sr1^iv^	125.435 (5)	O1^vii^—Sr1—Pd1^xi^	86.43 (3)
O1^ii^—Pd1—Sr1^iv^	54.565 (5)	O1—Sr1—Pd1^xii^	125.435 (5)
Sr1^iii^—Pd1—Sr1^iv^	180.0	O2^xi^—Sr1—Pd1^xii^	86.845 (8)
O2^i^—Pd1—Sr1^v^	52.455 (4)	O2^xii^—Sr1—Pd1^xii^	37.546 (3)
O2—Pd1—Sr1^v^	127.545 (3)	O1^viii^—Sr1—Pd1^xii^	97.52 (3)
O1—Pd1—Sr1^v^	125.435 (5)	O1^iii^—Sr1—Pd1^xii^	86.43 (3)
O1^ii^—Pd1—Sr1^v^	54.565 (5)	O1^ix^—Sr1—Pd1^xii^	147.95 (5)
Sr1^iii^—Pd1—Sr1^v^	70.871 (10)	O1^vii^—Sr1—Pd1^xii^	38.82 (5)
Sr1^iv^—Pd1—Sr1^v^	109.129 (10)	Pd1^xi^—Sr1—Pd1^xii^	65.481 (6)
O2^i^—Pd1—Sr1^vi^	127.545 (3)	O1—Sr1—Pd1^xiii^	125.435 (5)
O2—Pd1—Sr1^vi^	52.455 (4)	O2^xi^—Sr1—Pd1^xiii^	86.845 (9)
O1—Pd1—Sr1^vi^	125.435 (5)	O2^xii^—Sr1—Pd1^xiii^	37.546 (3)
O1^ii^—Pd1—Sr1^vi^	54.565 (5)	O1^viii^—Sr1—Pd1^xiii^	38.82 (5)
Sr1^iii^—Pd1—Sr1^vi^	114.520 (6)	O1^iii^—Sr1—Pd1^xiii^	147.95 (5)
Sr1^iv^—Pd1—Sr1^vi^	65.480 (6)	O1^ix^—Sr1—Pd1^xiii^	86.43 (3)
Sr1^v^—Pd1—Sr1^vi^	75.090 (7)	O1^vii^—Sr1—Pd1^xiii^	97.52 (3)
O2^i^—Pd1—Sr1^vii^	52.455 (3)	Pd1^xi^—Sr1—Pd1^xiii^	109.130 (10)
O2—Pd1—Sr1^vii^	127.545 (3)	Pd1^xii^—Sr1—Pd1^xiii^	75.091 (7)
O1—Pd1—Sr1^vii^	54.565 (5)	O1—Sr1—Pd1^xiv^	125.435 (5)
O1^ii^—Pd1—Sr1^vii^	125.435 (5)	O2^xi^—Sr1—Pd1^xiv^	37.546 (3)
Sr1^iii^—Pd1—Sr1^vii^	65.480 (5)	O2^xii^—Sr1—Pd1^xiv^	86.845 (8)
Sr1^iv^—Pd1—Sr1^vii^	114.520 (6)	O1^viii^—Sr1—Pd1^xiv^	86.43 (3)
Sr1^v^—Pd1—Sr1^vii^	104.910 (7)	O1^iii^—Sr1—Pd1^xiv^	97.52 (3)
Sr1^vi^—Pd1—Sr1^vii^	180.0	O1^ix^—Sr1—Pd1^xiv^	38.82 (5)
O2^i^—Pd1—Sr1^viii^	127.545 (3)	O1^vii^—Sr1—Pd1^xiv^	147.95 (5)
O2—Pd1—Sr1^viii^	52.455 (3)	Pd1^xi^—Sr1—Pd1^xiv^	75.091 (7)
O1—Pd1—Sr1^viii^	54.565 (5)	Pd1^xii^—Sr1—Pd1^xiv^	109.130 (10)
O1^ii^—Pd1—Sr1^viii^	125.435 (5)	Pd1^xiii^—Sr1—Pd1^xiv^	65.481 (6)
Sr1^iii^—Pd1—Sr1^viii^	109.129 (10)	O1—Sr1—Sr1^xv^	90.0
Sr1^iv^—Pd1—Sr1^viii^	70.871 (10)	O2^xi^—Sr1—Sr1^xv^	133.011 (5)
Sr1^v^—Pd1—Sr1^viii^	180.0	O2^xii^—Sr1—Sr1^xv^	46.991 (5)
Sr1^vi^—Pd1—Sr1^viii^	104.910 (7)	O1^viii^—Sr1—Sr1^xv^	48.500 (3)
Sr1^vii^—Pd1—Sr1^viii^	75.090 (7)	O1^iii^—Sr1—Sr1^xv^	131.500 (4)
O2^i^—Pd1—Sr1^ix^	127.545 (4)	O1^ix^—Sr1—Sr1^xv^	131.500 (4)
O2—Pd1—Sr1^ix^	52.455 (3)	O1^vii^—Sr1—Sr1^xv^	48.500 (4)
O1—Pd1—Sr1^ix^	54.565 (5)	Pd1^xi^—Sr1—Sr1^xv^	122.741 (3)
O1^ii^—Pd1—Sr1^ix^	125.435 (5)	Pd1^xii^—Sr1—Sr1^xv^	57.260 (3)
Sr1^iii^—Pd1—Sr1^ix^	75.090 (7)	Pd1^xiii^—Sr1—Sr1^xv^	57.260 (3)
Sr1^iv^—Pd1—Sr1^ix^	104.910 (7)	Pd1^xiv^—Sr1—Sr1^xv^	122.741 (3)
Sr1^v^—Pd1—Sr1^ix^	114.520 (6)	Pd1—O1—Sr1	180.0
Sr1^vi^—Pd1—Sr1^ix^	70.871 (10)	Pd1—O1—Sr1^viii^	86.61 (5)
Sr1^vii^—Pd1—Sr1^ix^	109.129 (10)	Sr1—O1—Sr1^viii^	93.39 (5)
Sr1^viii^—Pd1—Sr1^ix^	65.480 (6)	Pd1—O1—Sr1^iii^	86.61 (5)
O2^i^—Pd1—Sr1^x^	52.455 (3)	Sr1—O1—Sr1^iii^	93.39 (5)
O2—Pd1—Sr1^x^	127.545 (3)	Sr1^viii^—O1—Sr1^iii^	173.23 (10)
O1—Pd1—Sr1^x^	125.435 (5)	Pd1—O1—Sr1^vii^	86.61 (5)
O1^ii^—Pd1—Sr1^x^	54.565 (5)	Sr1—O1—Sr1^vii^	93.39 (5)
Sr1^iii^—Pd1—Sr1^x^	104.910 (7)	Sr1^viii^—O1—Sr1^vii^	96.597 (9)
Sr1^iv^—Pd1—Sr1^x^	75.090 (7)	Sr1^iii^—O1—Sr1^vii^	83.001 (8)
Sr1^v^—Pd1—Sr1^x^	65.480 (5)	Pd1—O1—Sr1^ix^	86.61 (5)
Sr1^vi^—Pd1—Sr1^x^	109.129 (10)	Sr1—O1—Sr1^ix^	93.39 (5)
Sr1^vii^—Pd1—Sr1^x^	70.871 (10)	Sr1^viii^—O1—Sr1^ix^	83.001 (8)
Sr1^viii^—Pd1—Sr1^x^	114.520 (6)	Sr1^iii^—O1—Sr1^ix^	96.597 (9)
Sr1^ix^—Pd1—Sr1^x^	180.0	Sr1^vii^—O1—Sr1^ix^	173.23 (10)
O1—Sr1—O2^xi^	136.990 (5)	Pd1^xvi^—O2—Pd1	180.0
O1—Sr1—O2^xii^	136.990 (5)	Pd1^xvi^—O2—Sr1^ix^	90.0
O2^xi^—Sr1—O2^xii^	86.020 (11)	Pd1—O2—Sr1^ix^	90.0
O1—Sr1—O1^viii^	86.61 (5)	Pd1^xvi^—O2—Sr1^iv^	90.0
O2^xi^—Sr1—O1^viii^	119.68 (4)	Pd1—O2—Sr1^iv^	90.0
O2^xii^—Sr1—O1^viii^	65.87 (4)	Sr1^ix^—O2—Sr1^iv^	180.0
O1—Sr1—O1^iii^	86.61 (5)	Pd1^xvi^—O2—Sr1^viii^	90.0
O2^xi^—Sr1—O1^iii^	65.87 (4)	Pd1—O2—Sr1^viii^	90.0
O2^xii^—Sr1—O1^iii^	119.68 (4)	Sr1^ix^—O2—Sr1^viii^	86.018 (11)
O1^viii^—Sr1—O1^iii^	173.22 (10)	Sr1^iv^—O2—Sr1^viii^	93.982 (11)
O1—Sr1—O1^ix^	86.61 (5)	Pd1^xvi^—O2—Sr1^vi^	90.0
O2^xi^—Sr1—O1^ix^	65.87 (4)	Pd1—O2—Sr1^vi^	90.0
O2^xii^—Sr1—O1^ix^	119.68 (4)	Sr1^ix^—O2—Sr1^vi^	93.982 (11)
O1^viii^—Sr1—O1^ix^	83.000 (8)	Sr1^iv^—O2—Sr1^vi^	86.018 (11)
O1^iii^—Sr1—O1^ix^	96.597 (9)	Sr1^viii^—O2—Sr1^vi^	180.0

Symmetry codes: (i) *x*, *y*−1, *z*; (ii) −*x*+1, −*y*, −*z*+1; (iii) −*x*+1/2, −*y*−1/2, −*z*+1/2; (iv) *x*+1/2, *y*+1/2, *z*+1/2; (v) *x*−1/2, *y*−1/2, *z*+1/2; (vi) *x*−1/2, *y*+1/2, *z*+1/2; (vii) −*x*+3/2, −*y*−1/2, −*z*+1/2; (viii) −*x*+3/2, −*y*+1/2, −*z*+1/2; (ix) −*x*+1/2, −*y*+1/2, −*z*+1/2; (x) *x*+1/2, *y*−1/2, *z*+1/2; (xi) *x*−1/2, *y*−1/2, *z*−1/2; (xii) *x*+1/2, *y*−1/2, *z*−1/2; (xiii) *x*+1/2, *y*+1/2, *z*−1/2; (xiv) *x*−1/2, *y*+1/2, *z*−1/2; (xv) *x*+1, *y*, *z*; (xvi) *x*, *y*+1, *z*.
